# Severe Congenital Thrombocytopenia Characterized by Decreased Platelet Sialylation and Moderate Complement Activation Caused by Novel Compound Heterozygous Variants in *GNE*

**DOI:** 10.3389/fimmu.2021.777402

**Published:** 2021-11-09

**Authors:** Karolina I. Smolag, Marcus Fager Ferrari, Eva Zetterberg, Eva Leinoe, Torben Ek, Anna M. Blom, Maria Rossing, Myriam Martin

**Affiliations:** ^1^ Section of Medical Protein Chemistry, Department of Translational Medicine, Lund University, Malmö, Sweden; ^2^ Clinical Coagulation Research Unit, Department of Translational Medicine, Lund University, Malmö, Sweden; ^3^ Department of Hematology, Rigshospitalet, Copenhagen University Hospital, Copenhagen, Denmark; ^4^ Children’s Cancer Center, Queen Silvia Children’s Hospital, Gothenburg, Sweden; ^5^ Center for Genomic Medicine, Rigshospitalet, Copenhagen University Hospital, Copenhagen, Denmark

**Keywords:** thrombocytopenia, GNE, factor H, complement activation, sialic acid, sialylation, high-throughput nucleotide sequencing

## Abstract

**Background:**

Hereditary thrombocytopenias constitute a genetically heterogeneous cause of increased bleeding. We report a case of a 17-year-old boy suffering from severe macrothrombocytopenia throughout his life. Whole genome sequencing revealed the presence of two compound heterozygous variants in *GNE* encoding the enzyme UDP-*N*-acetyl-glucosamine-2-epimerase/*N*-acetylmannosamine kinase, crucial for sialic acid biosynthesis. Sialic acid is required for normal platelet life span, and biallelic variants in *GNE* have previously been associated with isolated macrothrombocytopenia. Furthermore, sialic acid constitutes a key ligand for complement factor H (FH), an important inhibitor of the complement system, protecting host cells from indiscriminate attack.

**Methods:**

Sialic acid expression and FH binding to platelets and leukocytes was evaluated by flow cytometry. The binding of FH to erythrocytes was assessed indirectly by measuring the rate of complement mediated hemolysis. Complement activation was determined by measuring levels of C3bBbP (alternative pathway), C4d (classical/lectin pathway) and soluble terminal complement complex assays.

**Results:**

The proband exhibited markedly decreased expression of sialic acid on platelets and leukocytes. Consequently, the binding of FH was strongly reduced and moderate activation of the alternative and classical/lectin complement pathways was observed, together with an increased rate of erythrocyte lysis.

**Conclusion:**

We report two previously undescribed variants in *GNE* causing severe congenital macrothrombocytopenia in a compound heterozygous state, as a consequence of decreased platelet sialylation. The decreased sialylation of platelets, leukocytes and erythrocytes affects the binding of FH, leading to moderate complement activation and increased hemolysis.

## 1 Introduction

Inherited thrombocytopenias constitute a genetically heterogeneous group of disorders, associated with bleeding diathesis of variable severity (1). The increasing availability of high-throughput DNA sequencing (next-generation sequencing; NGS) has significantly improved the diagnostics of inherited thrombocytopenias and facilitated the identification of novel genes implicated in the pathogenesis (2–4). Currently, well over 30 different genes are known to harbor variants causative of inherited thrombocytopenias (5).

*GNE* encodes UDP-*N*-acetyl-glucosamine-2-epimerase/*N*-acetylmannosamine kinase, which is the rate-limiting enzyme of sialic acid biosynthesis (6). Sialic acids are expressed on all mammalian cells and have multifarious important biological roles in cell-cell interactions and immunity (7). *N*-acetylneuraminic acid (Neu5Ac) is the most abundant form of sialic acid and occupies the terminal position of glycoconjugates. On platelets, the presence of sialic acid is crucial for the normal life span, illustrated by the fact that sialic acid removal from the surface effectively triggers platelet clearance *via* the hepatic Ashwell-Morell receptor (8, 9).

On leukocytes, sialic acids are essential ligands for selectins and sialic acid-binding Ig-like lectins (siglecs), which play an important role in leukocyte recruitment and hence the regulation of immunity and inflammatory processes (10).

Sialic acids additionally regulate fluid phase innate immunity, as they are crucial for the ability of complement factor H (FH) to bind to hematopoietic cells. This occurs both by direct interaction, but more importantly, by enhancing the binding of FH to surface-deposited C3b thus inhibiting complement activation and protecting erythrocytes from complement mediated lysis (11). The complement system is a pivotal part of innate immunity and crucial for the defense against both microbial intruders and endogenous danger. Because of its destructive potential, complement is tightly controlled by inhibitors to avoid inflammation and tissues damage. FH is the main inhibitor of the alternative pathway, which is continuously activated in plasma but also in an amplification loop of the classical and lectin pathways. Genetic variants as well as autoantibodies affecting the C-terminal domains of FH impair the simultaneous binding of FH to sialic acid and C3b and are associated with complement-related diseases such as atypical hemolytic uremic syndrome, age-related macular degeneration and C3 glomerulopathy (12, 13). Decreased levels of the FH ligand sialic acid might result in the same outcome. Additionally, alternative pathway induced tissue destruction might in turn activate the classical and lectin pathways.

Deleterious variants in *GNE* have previously been associated with GNE myopathy, a rare autosomal recessive neuromuscular disorder characterized by progressive muscle wasting (14). Although not a hallmark symptom, congenital thrombocytopenia in patients suffering from GNE myopathy has previously been described in three unrelated families (15–17). Interestingly, isolated macrothrombocytopenia attributed to homozygous or compound heterozygous variants in *GNE* has just recently been reported in the literature (18–22). In addition, platelet sialylation was investigated in two of the reports, showing reduced expression of sialic acid on platelet surfaces (19, 20).

We report a rare case of severe congenital macrothrombocytopenia in an otherwise healthy 17-year-old male, harboring two compound heterozygous variants in *GNE*. We assess the functional consequences of these variants regarding the sialylation of hematopoietic cells and binding of the complement inhibitor FH, in addition to assessing activation of the complement system and the presence of complement mediated hemolysis.

## 2 Methods

### 2.1 Study Population and Ethics

Signed informed consent was obtained from the proband and his family members prior to the genetic investigation and further functional testing. Healthy controls were recruited at the Department of Translational Medicine, Lund University, Malmö. All samples were collected in accordance with the Declaration of Helsinki. The study was approved by the Ethics Committee, Lund University, Sweden (Dnr 2014/409, Dnr 2017/582).

### 2.2 Whole Genome Sequencing

Genomic DNA was purified from whole blood using a Nextera DNA Flex Library Preparation Kit (Illumina). Sequencing was performed using the NovaSeq 6000 platform (Illumina) at an average sequencing depth of at least 30x, and 98% of the genome was sequenced at least x10. The resulting fastq files were mapped to the hg19/GRCh37 human reference genome. Germline variant calling was performed using GATK v3.8.0, following Best Practices guidelines (23). The variants were filtered using Ingenuity Variant Analysis Software (Qiagen). Allele frequencies were derived from gnomAD v2.1.1 (https://gnomad.broadinstitute.org) (24).

### 2.3 Transmission Electron Microscopy

Platelet morphology was assessed in the proband by transmission electron microscopy using the whole mount method (25). Platelet rich plasma (PRP) was prepared as previously described (26) and was added on a carbon coated mesh grid, subsequently rinsed with distilled water and left to air-dry. The samples were examined using a CM 100 transmission electron microscope (Phillips/FEI) operated at 100 kV accelerating tension. Images were recorded with a Veleta camera system and the iTEM FEI software v5.1 package (both Olympus Soft Imaging Solutions).

### 2.4 Mean Platelet Volume and Immature Platelet Fraction

The mean platelet volume and the immature platelet fraction was measured on a Sysmex XN-10 automated hematology analyzer (Sysmex Corporation).

### 2.5 Sialylation and FH Binding on Platelets and Leukocytes

#### 2.5.1 Isolation of Platelets From Whole Blood

Whole blood was carefully drawn into Vacutainer sodium citrate tubes. PRP was obtained after centrifugation for 15 min at 250 × g and transferred into ACD buffer (38 mM anhydrous citric acid, 75 mM trisodium citrate dihydrate, 124 mM glucose monohydrate, pH 6.5), which was freshly supplemented with 2 mM adenosine and 7.5 mM theophylline (ACD+A/T). Platelets were washed twice with fresh buffer by centrifugation for 10 min at 1000 × g. Platelet count measurements were performed using Flow-Count Fluorospheres (Beckman Coulter) and platelets were diluted with ACD+A/T buffer to a final concentration of 1 x 10^7^/mL.

#### 2.5.2 Detection of Sialic Acid on Platelets and Leukocytes

Platelets were gated using CD42b-APC and CD61-PE (#551061, #IM3605; Beckman Coulter) antibodies. For the isolation of leukocytes, whole blood was drawn into Vacutainer EDTA tubes. Erythrocytes were lysed using a TQ-Prep Workstation (Beckman Coulter) and the remaining cells were washed with PBS. Lymphocytes and monocytes were gated using CD5-PC5.5 and CD14-PC7 antibodies (#B49191, #A22331; Beckman Coulter), respectively. After exclusion of lymphocytes and monocytes, granulocytes were gated by forward (FSC) and side scatter (SSC) characteristics. α-2,6- and α-2,3-linked sialic acid was detected using 250 ng/mL Cy5-labeled *Sambucus nigra* lectin (SNA) (#CL-1305; Vector Laboratories) and 100 μg/mL FITC-labeled *Maackia amurensis* lectin II (MAL II) (#21511103-1; GlycoMatrix), respectively. Cells were acquired on a CytoFLEX flow cytometer (Beckman Coulter) and analyzed with FlowJo software (Tree Star). To adjust for distinct autofluorescent properties of investigated cells, results were presented as delta geometric mean fluorescence intensity (Δ gMFI) in relation to buffer treated cells.

#### 2.5.3 Assessment of FH Binding in Platelets and Leukocytes

Washed platelets and leukocytes were incubated with 175 μg/mL DyLight405 (DL405)-labeled FH in binding buffer (10 mM HEPES, 150 mM NaCl, 5 mM KCl, 1 mM MgCl_2_) in the presence of 2% (platelets) or 10% (leukocytes) normal human serum (NHS) as a source of C3b. The serum was pre-incubated with 25 μg/mL *Ornithodoros moubata* salivary protein (OmCI) a potent inhibitor of C5 convertases to prevent cell lysis (27). Cells were acquired and analyzed as above.

### 2.6 Hemolytic Assay

Erythrocytes were carefully washed at 4°C using veronal buffered saline with 0.1% gelatin, 1 mM MgCl_2_, 0.15 mM CaCl_2_ and 2.5% dextrose (DGVB++) and diluted to a final concentration of 5 x 10^8^ cells/mL. Erythrocytes were incubated for 1 h at 37°C with freshly frozen serum in increasing concentrations from 2.5% to 30%. Free hemoglobin was measured by absorbance at 405 nm in the supernatants after removal of erythrocytes by centrifugation for 3 min at 800 × g. Absorbance was measured using a Cytation 5 Multi-Mode Reader (BioTek Instruments). An anti-erythrocyte antibody (#ab197770; Abcam) was added together with 10% serum to confirm the possibility of complement mediated hemolysis.

### 2.7 Complement C3bBbP, C4d, and sTCC

Plasma levels of C3bBbP, C4d and soluble terminal complement complex (sTCC) were measured using enzyme-linked immunosorbent assays (ELISAs). The Complement C4d (#COMPL C4d RUO) and Complement TCC (#COMPL TCC RUO; both Svar Life Science) assays were used according to the manufacturer’s instruction. Quantification of C3bBbP complexes in plasma from the proband, parents and healthy controls (*n* = 31) was carried out using a homemade assay: Microtiter plates were coated overnight with mouse anti-properdin antibodies (#A235; Quidel) to trap the complexes and unbound antibodies were removed by washing with PBS supplemented with 0.2% Tween-20 (PBST). Plasma samples were incubated for 1 h at 4°C. C3bBbP complexes were detected using rabbit anti-human C3c antibodies (#A0062; Dako), followed by addition of peroxidase labelled swine anti-rabbit secondary antibodies (#P0399; Dako). TMB One substrate (#4380A; Kementec) was added and the reaction was stopped with 0.5 M H2SO4. PBST was used for washing in-between all steps. Absorbance was measured in Cytation 5 Multi-Mode Reader (BioTek Instruments). The local reference intervals of normal are 38 – 196 mg/mL for C4d (*n* = 50), 6.5 – 23.4 CAU/mL for C3bBbP (*n* = 31) and 31 – 131 mg/mL for sTCC (*n* = 50).

### 2.8 Statistics

For the flow cytometrical experiments, the median Δ gMFI value is presented together with sample minimum and maximum (min-max) in the control groups. The results in the proband and the parents are presented as the percentage of the median Δ gMFI value in the control group for each experiment. For the hemolytic assays, all values are presented as the increase in absorbance from baseline (0% serum). The results in the control group are presented as the median values together with sample maximum (max). Data were analyzed and graphed using GraphPad Prism v9.0.2 for Mac OS X (GraphPad Software).

## 3 Results

### 3.1 Clinical Characteristics and Family Description

The proband is a currently 17-year-old Caucasian male suffering from severe macrothrombocytopenia since birth, with platelet counts ranging from <5 x 10^9^/L to 10 x 10^9^/L. At delivery, he was diagnosed with intracranial hemorrhage following vacuum extraction and throughout the years he exhibited frequent episodes of spontaneous mucosal bleeding due to the low platelet counts. Erythrocyte and platelet transfusions were required on a few occasions due to the severity of the bleeds. Furthermore, infectious episodes did always result in an increased bleeding tendency. At the age of 11 years, he suffered from prolonged fever and lymphadenopathy together with extensive intravascular hemolysis causing nocturnal hemoglobinuria. The direct antiglobulin test (DAT) was negative. Lymph node biopsy confirmed the presence of Kikuchi-Fujimoto disease, that was self-resolving (28).

**Figure 1:**
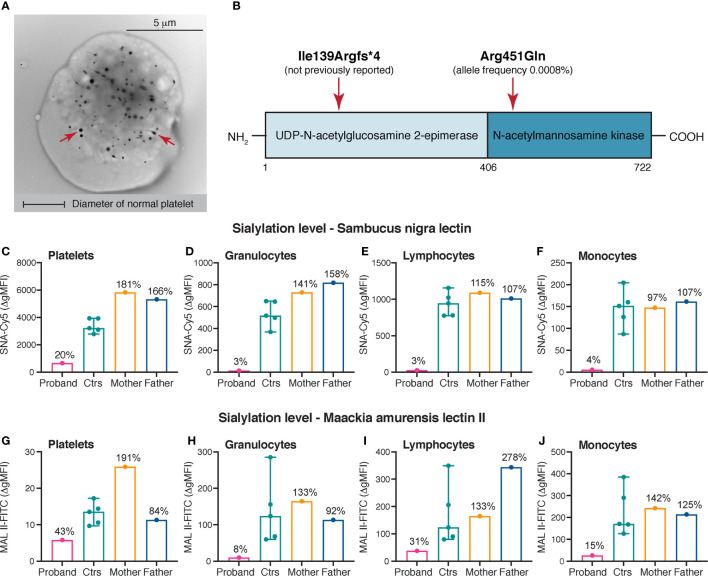
Platelet electron microscopy, variants identified in *GNE* and sialylation levels on platelets and leukocytes. **(A)** Whole mount transmission electron microscopy image of a giant platelet (> 8 μm in diameter) of the proband. Original magnification x7.900. For comparison the diameter of a normal platelets is depicted. The red arrows mark dense granules. **(B)** Linear domain organization of UDP-*N*-acetyl-glucosamine-2-epimerase/*N*-acetylmannosamine kinase encoded by *GNE.* The approximate positions of the variants identified in the proband are shown. **(C–J)** Sialylation of platelets, granulocytes, lymphocytes and monocytes. α-2,6- and α-2,3-linked sialic acid was detected by flow cytometry using Cy5-labeled SNA (250 ng/mL) and FITC-labeled MAL II (100 μg/mL), respectively. A markedly decreased sialylation of all cell types was demonstrated in the proband compared with healthy controls. The heterozygous parents of the proband did not show reduced levels of sialic acid on the investigated cell types. The results of the healthy controls (*n* = 5) are shown as medians with min-max. The results in the proband and the parents are presented as percentages of the median Δ gMFI in the control group. SNA, Sambucus nigra lectin; MAL II, Maackia amurensis lectin II; gMFI, geometric mean fluorescence intensity.

At the age of 12 years, the mean platelet volume was 18 femtoliters (reference range: 9.4 - 12.6 femtoliters) and investigation by transmission electron microscopy showed giant platelets >10 μm in diameter (**Figure 1A**). An increased mean platelet size (forward scatter) was also confirmed by flow cytometry (data not shown). Flow cytometry showed normal expression of platelet GPIb, excluding Bernard-Soulier syndrome. Furthermore, thrombocytopenia related to telomere length, *GATA1* and *MYH9*-variants was ruled out. The white blood cell differential was always normal, with the exception of a persistent mild neutropenia with neutrophil counts varying between 0.50 x 10^9^/L and 1.00 x 10^9^/L. During the present investigation, neutrophil counts ranged from 0.93 x 10^9^/L to 1.38 x 10^9^/L (reference range: 1.70 - 7.50 x 10^9^/L). The immature platelet fraction was significantly elevated, ranging from 45% to 72% (reference range: 1.1 - 6.1%), consistent with previous reports on *GNE*-associated thrombocytopenia (18, 19). Repeated bone marrow aspirates and trephines showed normal findings, including an average number of non-dysmorphic megakaryocytes. The spleen size was in the normal range when investigated by abdominal ultrasound. Importantly, the proband did not show any signs of myopathy. The first-degree relatives of the proband (mother, father and brother) were healthy, with normal platelet counts. No history of thrombocytopenia or other hematological diseases was evident in the family.

### 3.2 Presence of Two Compound Heterozygous Variants in *GNE*

Whole genome sequencing was undertaken in the proband and his first-degree relatives. In the proband, genetic screening revealed the presence of two heterozygous variants in *GNE* (c.416_426del, p.Ile139Argfs*4 and c.1352G>A, p.Arg451Gln) (**Figure 1B**). The exon 4 deletion was not previously reported, while the exon 8 missense variant was found to be extremely rare with a total allele frequency 0.0008% using the gnomAD browser (24). In addition, a hemizygous missense variant in *FLNA* (c.2449C>T, p.Pro817Ser) located to the X-chromosome and a heterozygous missense variant in *SLFN14* (c.1358A>G, p.Asn453Ser) was identified in the proband, both assessed as nonsignificant following immunohistochemistry (*FLNA*) and whole mount platelet electron microscopy (*SLFN14*) (29, 30). The variants in *GNE* were present in a compound heterozygous state in the proband; the p.Ile139Argfs*4 variant was inherited from the mother and the p.Arg451Gln variant was inherited from the father. The brother of the proband did only inherit the paternal variant.

### 3.3 Decreased Sialylation of Platelets and Leukocytes

Levels of sialic acid on platelets and leukocytes were assessed in the proband, the parents and healthy controls. The suspected pathogenicity of the compound heterozygosity for the *GNE* variants was confirmed by drastically decreased levels of sialic acid on platelets (SNA: 20%, MAL II: 43%), granulocytes (SNA: 3%, MAL II: 8%), lymphocytes (SNA: 3%, MAL II: 31%) and monocytes (SNA: 4%, MAL II: 15%) in comparison to healthy controls (SNA and MAL II: 100%) (**Figures 1C–J**). The heterozygous parents showed normal results, suggesting a sufficient residual activity of the GNE enzyme for sialic acid biosynthesis (**Figures 1C–J**).

### 3.4 Decreased Binding of Complement Inhibitor FH on Platelets and Leukocytes

**Figure 2:**
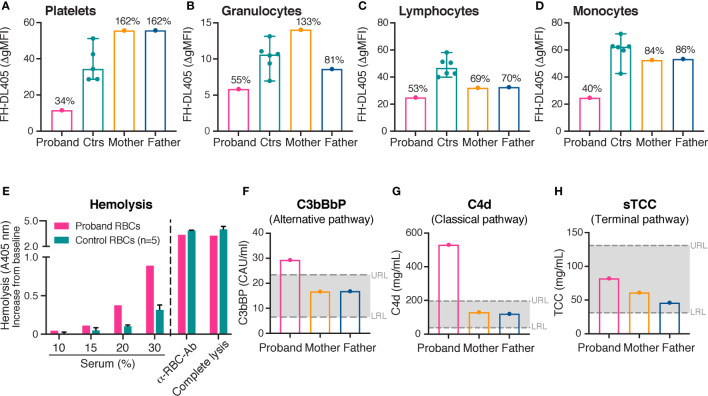
Binding of complement inhibitor FH on platelets and leukocytes, hemolytic assay and complement activation. **(A–H)** FH binding to platelets **(A)**, granulocytes **(B)**, lymphocytes **(C)** and monocytes **(D)**. Binding of FH was investigated by flow cytometry using purified DyLight 405-labeled FH (175 μg/mL). A decreased binding of FH to all cell types was demonstrated in the proband compared with healthy controls. The heterozygous parents showed normal binding of FH to platelets, granulocytes and monocytes; FH binding to lymphocytes was however slightly reduced compared with healthy controls. The results of the healthy controls (platelets, *n* = 5; granulocytes, lymphocytes and monocytes, *n* = 6) are shown as medians with min-max. The results in the proband and the parents are presented as percentages of the median Δ gMFI in the control group. **(E)** Assessment of hemolysis. Washed erythrocytes from the proband and healthy controls were incubated with normal human serum in increasing concentrations from 10% to 30%. An anti-erythrocyte antibody was added to 10% serum to confirm the possibility of complement mediated hemolysis. For comparison, complete lysis (erythrocytes treated with H_2_O) is shown. Free hemoglobin was measured by absorbance at 405 nm. The increase in absorbance from baseline (0% serum) is shown on the Y-axis. An increased rate of hemolysis was observed in the proband compared with healthy controls. The results of the healthy controls (*n* = 5) are shown as medians with max. **(F–H)** Levels of alternative (C3bBbP), classical/lectin (C4d) and terminal complement pathway (sTCC) activation. A moderate activation of the alternative and the classical/lectin complement pathways was observed in the proband, but not in his parents. gMFI, geometric mean fluorescence intensity; FH, complement inhibitor factor H; RBC, erythrocyte; Ab, antibody; A, absorbance; URL, upper reference limit; LRL, lower reference limit.

FH binding to platelets and leukocytes was assessed in the proband, the parents and healthy controls. To resemble the physiological situation, serum was added as a source of deposited C3b to fully assess the effect of desialylation on FH binding. Consistent with the decreased sialylation, a markedly reduced binding of FH to platelets (34%), granulocytes (55%), lymphocytes (53%) and monocytes (40%) was shown in the proband compared with healthy controls (100%) (**Figures 2A–D**). The heterozygous parents showed normal binding of FH to platelets, granulocytes and monocytes. However, the binding of FH to lymphocytes was slightly reduced compared with healthy control (**Figure 2C**).

### 3.5 Increased Erythrocyte Lysis

Hemolytic assays were performed in the proband and healthy controls. The erythrocytes of the proband showed an increased rate of lysis compared with healthy controls (**Figure 2E**). The results were independent of whether erythrocytes were incubated with serum from the proband or healthy controls, excluding an interfering factor present in the serum of the proband (data not shown).

### 3.6 Moderate Complement Activation

Plasma levels of C3bBbP, a marker for alternative complement pathway activation; C4d, a marker for classical and lectin pathway activation; and sTCC, a marker for terminal complement pathway activation were measured in the proband and the parents at the time point of this study. Levels of circulating C3bBbP (**Figure 2F**) and C4d (**Figure 2G**) were moderately elevated in the proband in comparison to healthy controls. However, levels of sTCC (**Figure 2H**) were observed at normal levels.

### 3.7 Clinically Insignificant Alteration in Platelet Counts During Treatment With Oseltamivir and Eltrombopag

**Figure 3:**
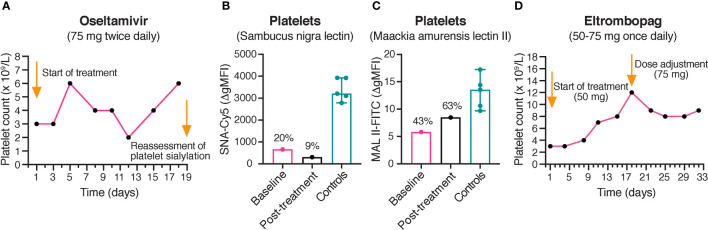
Platelet counts during treatment with oseltamivir and eltrombopag. **(A)** Platelet counts in the proband during treatment with oseltamivir. The platelet counts of the proband were monitored during treatment with oseltamivir 75 mg twice daily. No clinically significant increase of the platelet count was observed after 17 days of treatment. **(B, C)** Reassessment of platelet sialylation after treatment with oseltamivir. α-2,6- and α-2,3-linked sialic acid was detected by flow cytometry using Cy5-labeled SNA (250 ng/mL) and FITC-labeled MAL II (100 μg/mL), respectively. A persistently decreased sialylation of platelets was observed in the proband following 18 days of treatment with oseltamivir. The results of the healthy controls (*n* = 5) are shown as medians with min-max. The results in the proband are presented as percentages of the median Δ gMFI in the control group. **(D)** Platelet counts in the proband during treatment with eltrombopag. The platelet counts of the proband were monitored during treatment with eltrombopag 50-75 mg once daily. No clinically significant increase of the platelet count was observed after 31 days of treatment. SNA, Sambucus nigra lectin; MAL II, Maackia amurensis lectin II; gMFI, geometric mean fluorescence intensity.

Due to the severity of the patient’s thrombocytopenia, it was decided on clinical grounds to try off-label treatment with oseltamivir, an antiviral neuraminidase inhibitor used for treatment of influenza. Consequently, the proband was treated with oseltamivir 75 mg twice daily. Since we had ethic approval for blood sampling in order to functionally characterize the genetic variants, the effect on platelet count and platelet sialic acid was evaluated. After 17 days, the platelet count was only 6 x 10^9^/L (**Figure 3A**), and thus clinically not significantly increased. The levels of platelet sialic acid were persistently decreased after 18 days of treatment (**Figures 3B, C**). Thus, the effect of oseltamivir on endogenous sialidases *in vivo* could possibly be insufficient for rescuing sialic acid on the markedly desialylated platelets observed in the proband.

Subsequently, it was decided by the treating physician to try off-label treatment with the thrombopoietin (TPO) receptor agonist eltrombopag, since it has recently been reported to increase platelet counts in different forms of inherited thrombocytopenias (31). The proband was treated with eltrombopag 50-75 mg once daily. However, only a slight increase of the platelet count to 9 x 10^9^/L was observed following 31 days of treatment (**Figure 3D**).

## 4 Discussion

The present report shows evidence of compound heterozygous variants in *GNE* (p.Ile139Argfs*4 and p.Arg451Gln) causing constitutional macrothrombocytopenia, as a result of decreased platelet sialylation.

The significantly decreased sialylation of platelets evident in the compound heterozygous proband is compatible with increased peripheral clearance of platelets (8). Accordingly, a very high immature platelet fraction was observed, in line with previous reports on patients with *GNE*-associated thrombocytopenia (18, 19). The significance of the variants in *GNE* was further confirmed by a decreased sialylation of leukocytes. Indeed, the GNE enzyme is known to be an important determinant of normal sialylation in human hematopoietic cells (32).

Interestingly, a mild neutropenia was found in the proband. To our knowledge, no association between neutropenia and variants in *GNE* has previously been reported. However, variable neutropenia has previously been described in a patient with macrothrombocytopenia attributed to deficient α-2,3-sialylation observed in platelets and granulocytes (33). Taken together, the possibility of decreased sialylation contributing to the neutropenia observed in the proband cannot be ruled out. Normal levels of sialylation were demonstrated in the heterozygous mother (p.Ile139Argfs*4) and father (p.Arg451Gln) of the proband, suggesting a residual activity of the GNE enzyme sufficient for normal sialic acid biosynthesis when the variants are present in a heterozygous state.

Previously reported variants causative of *GNE*-associated thrombocytopenia have been located in the *N*-acetylmannosamine kinase domain of the UDP-*N*-acetyl-glucosamine-2-epimerase/*N*-acetylmannosamine kinase, except for one case of a compound heterozygous patient with a missense variant in exon 4 (c.562C>T, p.His188Tyr) (19). Hence, our results confirm the potential pathogenicity of deleterious variants located in the UDP-*N*-acetyl-glucosamine-2-epimerase domain, causing isolated macrothrombocytopenia in a recessive mode of inheritance. Biallelic variants in *GNE* are associated with GNE myopathy (34) and causative variants are known to be located in both the epimerase and kinase domains of the GNE enzyme (14).

Consistent with the decreased sialylation, a significantly reduced binding of FH to platelets and leukocytes was observed in the proband. The proband did not show any rare or significant variants in the *CFH* gene encoding FH, including age-related macular degeneration (AMD) related polymorphisms (Val62Ile, Tyr402His and Glu936Asp) (35–37). Hence, the reduced FH binding was attributed to the decreased sialylation shown in platelets and leukocytes. Moreover, an increased rate of hemolysis was evident in the proband, most likely explained by reduced binding of FH to erythrocytes (12). The hemolysis observed in the present study was likely subclinical, since the levels of haptoglobin (0.39 g/L, reference range: 0.30 - 2.00 g/L) and lactate dehydrogenase (3.9 μkat/L, reference range: 1.9 - 4.3 μkat/L) were normal.

Interestingly, at the age of 11 years, the proband suffered from a self-resolving episode of Kikuchi-Fujimoto disease. Kikuchi-Fujimoto has been associated with autoimmune conditions such as systemic lupus erythematosus, characterized by activation of the complement system (38). Interestingly, the proband showed moderately elevated levels of circulating C3bBbP and C4d, indicating that the episode could have been related to an increased complement activity due to a permanently reduced protection from complement activation by FH. Nevertheless, the present levels of sialic acid seem to be sufficient for FH to prevent an uncontrolled activation of complement, reflected by normal levels of sTCC. However, it is possible that the proband might develop future autoimmune or inflammatory conditions triggered by external factors, as evident from the episode of Kikuchi-Fujimoto disease (28).

Current treatment options for *GNE*-associated thrombocytopenia have been limited to platelet transfusions. Interestingly, platelet counts have been observed to increase in non-thrombocytopenic influenza patients treated with oseltamivir (39). Moreover, oseltamivir in combination with other therapies has recently been reported to increase platelet counts in therapy resistant immune thrombocytopenia (ITP), in which desialylation of platelets has been presented as an important pathophysiological mechanism (40). Oseltamivir as monotherapy was, however, ineffective. Importantly, although highly effective in inhibiting viral neuraminidases, oseltamivir has been shown to exhibit a low potency for human neuraminidases *in vitro* (41). This finding seems beneficial, since antivirals should have limited effects of human neuraminidases to avoid side effects. Interestingly, a SNP in human neuraminidase 2 increases its susceptibility to oseltamivir and is a concern for side effects in the Asian population (42).

After 17 days of treatment with oseltamivir, the platelet count was only 6 x 10^9^/L, consistent with persistently decreased platelet sialic acid levels. Thus, the low potency of oseltamivir on human sialidases *in vivo* was likely insufficient for rescuing the sialic acids on the probands platelets. In comparison to ITP, in which the desialylation is antibody-mediated, the platelets of the proband are already produced in a desialylated form due to a defective GNE enzyme. Only a slight increase of the platelet was observed following 31 days of treatment with eltrombopag, most likely due to rapid platelet clearance and pre-existing high endogenous TPO levels, since hepatic removal of desialylated platelets up-regulates hepatic TPO synthesis, possibly *via* the Ashwell-Morrell receptor (43).

Currently, the proband only experiences occasional mild bleeding symptoms. Interestingly, therapies aiming at delivering precursors in the sialic acid biosynthesis pathway are under development for the treatment of GNE myopathy: A phase 2 trial of ManNAc-administration to patients with GNE myopathy recently showed biochemical and clinical efficacy, together with long-term safety (44). Even though no results were reported regarding the effects on platelet sialylation, ManNAc could potentially be implemented as a treatment option for *GNE*-associated thrombocytopenia in the future.

In summary, we present two novel compound heterozygous variants in *GNE*, causing severe congenital macrothrombocytopenia as a result of decreased platelet sialylation. The decreased sialylation of platelets, leukocytes and erythrocytes was associated with reduced binding of the complement inhibitor FH, resulting in increased hemolysis and moderate complement activation.

## Data Availability Statement

The datasets presented in this article are not readily available because they are obtained to present a case report, where data cannot be totally anonymous. Moreover, since the proband is a child, special ethical considerations must be made. Requests to access the datasets should be directed to EZ, Department of Translational Medicine, Skane University Hospital, Malmö, Sweden, eva.zetterberg@med.lu.se.

## Ethics Statement

The study was approved by the Ethics Committee, Lund University, Sweden (Dnr 2014/409, Dnr 2017/582). Written informed consent to participate in this study was provided by the participants’ legal guardian/next of kin. Written informed consent was obtained from the individual(s), and minor(s)’ legal guardian/next of kin, for the publication of any potentially identifiable images or data included in this article.

## Author Contributions

EZ, MM, MR, EL, MFF, KS, and AB participated in the design of the study. EZ, MFF, and TE provided the clinical data. MR performed the genetic analyses. MM and KS developed and performed the flow cytometrical assays and the assays measuring hemolysis and complement activation. MFF performed the transmission electron microscopy. All authors participated in the interpretation of the results. MFF wrote the manuscript, which was critically reviewed by all authors. KIS and MFF contributed equally to this article as joint first authors. MR and MM contributed equally to this article as joint senior authors. All authors contributed to the article and approved the submitted version.

## Funding

The study was financed by governmental funding of clinical research within the NHS (National Health Services).

## Conflict of Interest

The authors declare that the research was conducted in the absence of any commercial or financial relationships that could be construed as a potential conflict of interest.

## Publisher’s Note

All claims expressed in this article are solely those of the authors and do not necessarily represent those of their affiliated organizations, or those of the publisher, the editors and the reviewers. Any product that may be evaluated in this article, or claim that may be made by its manufacturer, is not guaranteed or endorsed by the publisher.
